# Optimization of an analytical methodology to determine microplastic contamination in different seaweed groups (Phaeophyceae, Rhodophyta and Chlorophyta)

**DOI:** 10.1016/j.mex.2026.103816

**Published:** 2026-02-15

**Authors:** Rúben Pereira, C. Marisa R․ Almeida, Sandra Ramos

**Affiliations:** a Institute of Biomedical Sciences Abel Salazar (ICBAS), University of Porto, Rua de Jorge Viterbo Ferreira no 228, Porto 4050-313, Portugal; b Interdisciplinary Centre of Marine and Environmental Research (CIIMAR), University of Porto, Terminal de Cruzeiros do Porto de Leixões, Avenida General Norton de Matos, S/N, Matosinhos 4450-208, Portugal; c Chemistry and Biochemistry Department, Faculty of Sciences, University of Porto, Rua do Campo Alegre, 687, Porto 4169-007, Portugal; d Biology Department, Faculty of Sciences, University of Porto, Rua do Campo Alegre, s/n, Porto, 4169– 007, Portugal

**Keywords:** Seaweed, Digestion, Enzymatic, Oxidative, Method, Microplastics, Contamination

## Abstract

Seaweed are primary producers and potential vectors of microplastics (MPs) contamination, yet robust extraction methods that digest complex algal matrices while preserving polymer integrity remain limited. A sequential enzymatic–oxidative digestion was optimized for three seaweeds (*Fucus vesiculosus, Chondrus crispus* and *Ulva lactuca*). The optimized process involved the initial addition of cellulase (1% w/v, 24 h, 50 °C) followed by H₂O₂ (30% v/v, 48–72 h, 65 °C). Across nine 0.5 g dry-weight sub-replicates (3 per seaweed), 30 MPs were found (6.7 MPs/g⁻¹). The integrity of polymers was assessed for 12 MPs polymers, with acceptable performance being defined as ≥ 90% recovery and spectroscopic (through FTIR analysis) identifiability. Eight polymers met this threshold (90–101%). Four polymers were adversely affected with the long 72 H₂O₂ incubation, namely: cellulose-acetate (53% recovery), polyamide (61%), acrylic (3%) and rayon (2%). Although polymers remained identifiable, sequential digestion produced mass loss and visible changes (e.g. polyamide opacity, cellulose-acetate brittleness), which may increase fragmentation and miss-identification. Therefore, the protocol is suitable for most common MPs, but not for rayon and acrylic, and should be applied cautiously where cellulose-acetate or polyamide are expected.

## Abstract (author highlights)

• Methodology optimized to analyzed MPs contamination in three seaweed groups.
• A sequential enzymatic–oxidative digestion preserved most MPs integrity.
• Most MPs (8 out of 12) polymers had recovery rates > 90 %.

## Graphical abstract

**Figure:**
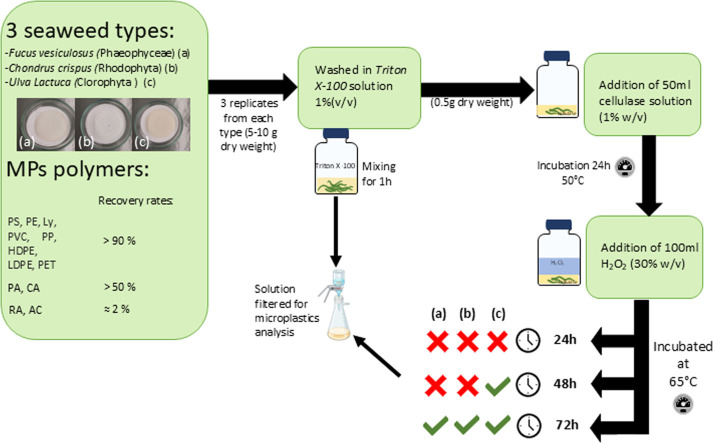

Specifications table

**Table:** 

**Subject area**	Environmental Science
**More specific subject area**	Microplastics pollution
**Name of your method**	Enzymatic-Oxidative digestion to quantify microplastics in various seaweed types
**Name and reference of original method**	López-Rosales et al. 2022 [1]
**Resource availability**	NA

## Background

Microplastics (MPs) constitute a concern to all ecosystems [2], and their presence was already reported in all types of marine organisms from smaller herbivores or planktonic organisms to larger organisms, MPs contamination occurring not only from direct ingestion but also through trophic transfer [3]. However, studies related to the presence of MPs at the basis of marine trophic chains are still not abundant. Most studies focus on planktonic organisms [4,5]. However, research related to seaweed has only started to develop in recent years [[6], [7], [8]], showing that these organisms are also contaminated with MPs. MPs contamination might negatively impact seaweeds and compromise the ecological and economic importance of seaweed [9].

Seaweed forms marine forests with extreme ecological importance [10,11]. As primary producers they are essential for marine oxygen production and carbon sequestration, they are also at the bottom of most marine food chains serving as food sources for a variety of marine organisms. Marine forests also provide essential refuge as well as nursery habitats for several marine organisms [11]. Furthermore, seaweeds are not only important at the ecosystem level, they also have high economic importance being used as a food source for humans [12,13], as well as being used as raw materials for cosmetics and pharmaceutical industries [11,14].

Seaweeds can retain MPs either by adsorption or absorption [6,[15], [16], [17]]. Several authors suggested that the electrostatic properties of MPs, cellulose content as well as biofilm presence play an important role in MPs adsorption to seaweed [1,7,13,15,16]. The existence of air vesicles in some seaweed (i.e. *Ulva lactuca*., *Fucus vesiculosus*) may also contribute to MPs absorption [18]. This ability of seaweed to adsorb and absorb MPs may have implications in MPs transfer across the food chain [7,16,19]. For instance, Gutow et al. [16] reported trophic transfer of MPs from *F. vesiculosus* to marine gastropods *Littorina littorea*. Furthermore, the adherence of MPs to seaweed may lead to the transference of other pollutants, such as additives (phthalates, BPA, etc.) and metals [20,21]. There is a growing concern regarding this issue since several species of algae are consumed directly by fish, as well as by humans [7,13,22]. However, more studies are needed to clarify all these processes.

Washing only approaches for MPs extraction from seaweed samples have unsatisfactory MPs recovery rates and are far from being standardized [1,16,17,19]. So, seaweed digestion should be carried out. Some studies employ varying methods to digest the organic matter of the seaweed [1,12,15,18]. However, the lack of harmonized methodologies makes comparability of results difficult and unreliable due to methodological differences [8,23].

As such, the methodology optimized in this study was specifically designed to infer the most efficient and cost-effective MPs extraction method from seaweed. The methodology aims to ensure both the complete elimination of seaweed tissues while trying to preserve the integrity of 12 polymers of MPs commonly found in environmental samples, for which their size, color, visual aspect as well as polymer characteristics were analyzed.

## Method details

### Contamination control

Since MPs are ubiquitous, it is crucial to ensure that no contamination of the samples occurs during all the procedures performed, and measures are taken during MPs studies. For this work, several precautions were taken to avoid contaminations, including: the use of glass materials and aluminum for samples storage/analysis; cleaning of all the materials used with ethanol and deionized water before use; when not in use material was covered in aluminum foil to avoid airborne contamination; a grey colored cotton laboratory coat was used at all steps to minimize fiber contamination from clothing; all solutions were checked for the presence of MPs under a stereomicroscope (Leica EZ04) and filtered through a through 0.45 μm pore size nitrate cellulose membrane filter when necessary; sample processing was done in a fume hood whenever possible.

To quantify possible sources of laboratory background contamination, blanks (i.e., a glass petri dish with filtered deionized water) were left exposed in the laboratory bench or fume hood where sampling processing occurred. Procedural blank samples with only filtered deionized water were subjected to the same methodology (digestion at selected temperatures and filtration) and inspected for MPs contamination at the end of the procedure as the samples. No MPs contamination was observed in any of the glass petri dishes nor in procedural blanks.

### Microplastics preparation

**Fig. 1:**
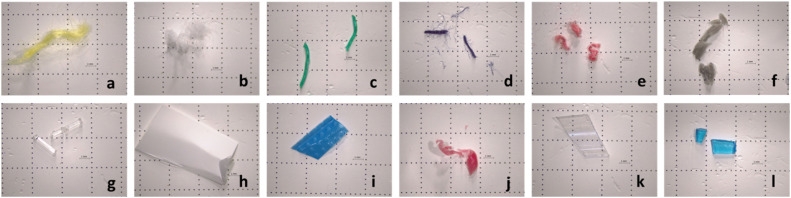
MPs prepared to be used in methodology optimization: (a) Acrylic; (b) Lycra; (c) Polyethylene; (d) Polystyrene; (e) Rayon; (f) Polyvinyl chloride; (g) Polyamide; (h) Cellulose acetate; (i) Polyethylene of low-density; (j) Polyethylene of high-density; (k) Polyethylene Terephthalate and (l) Polypropylene.

The types of MPs used in this research were chosen according to the most prevalent items and polymer types found in marine litter [2]. These MPs can come from different sources such as plastic bottles, clothing fabrics, fishing nets/ropes, cigarettes, among others. Although there are some studies regarding the effects of different digestion methodologies on MPs degradation (e.g. Rodrigues et al. [5,24], Silva et al. [25]), to the best of our knowledge only one study analyzed visual degradation of MPs when subjected to enzymatic digestion methodology and only 6 polymers were tested (PS, PP, PE, PET, PA, PVC) [1]. Moreover, in [16], the recovery rates were assessed by counting recovered items and polymers were only visually inspected. In the present study, tests were performed on 12 different polymers: polystyrene (PS), acrylic (AC), cellulose acetate (CA), lycra (Ly), polyamide (PA), polyvinyl chloride (PVC), polypropylene (PP), polyethylene (PE), polyethylene terephthalate (PET), polyethylene of high-density (HDPE), polyethylene of low-density (LDPE), rayon (RA) (Fig. 1). The MPs used were obtained by cutting manually larger plastic products, such as fabrics, bottles, fishing nets, PVC pipes, etc., and sieved through a 5 mm mesh to discard particles > 5 mm. MPs used were not brand new to better simulate MPs found in the environment.

To understand the effects of the methodology used on the studied MPs, MPs characteristics were recorded before and after the tests, by visual analysis of color, range of sizes and aspect (e.g., shape, appearance of the MP, fiber tightness), and by Fourier Transform Infrared Spectroscopy (FTIR) analysis to identify the polymer. Polymer spectra were registered in a PerkinElmer (Waltham, MA, USA) FTIR Spectrum 2 instrument coupled with attenuated total reflectance (ATR). Polymer spectra were determined after four scans at a wavelength range of 400 - 4000 cm^-1^. For polymer confirmation, the obtained spectra were compared with reference databases, and results with confidence levels > 75 % were accepted.

### Methodology optimization

**Fig. 2:**
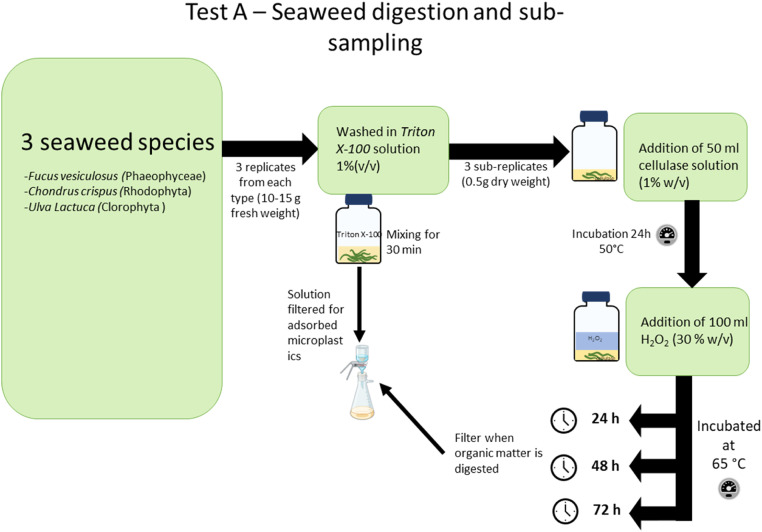
Representation of test A performed on three different seaweed species from the 3 main seaweed groups, Phaeophyceae (F. vesiculosus), Rhodophyta (C. crispus) and Clorophyta (U. lactuca), to optimize the seaweed tissues digestion methodology.
ii) Tests B.1 were performed with all selected MPs with two different steps, one incubation at 50 °C with a cellulase solution for 24 h followed by one of three different incubation periods (24, 48 or 72 h) at 65 °C with H_2_O_2_ (Fig. 3);

Three different types of tests were performed:

i) Tests A were performed with seaweeds samples from the 3 main seaweed groups Phaeophyceae (*F. vesiculosus*), Rhodophyta (*Chondrus crispus*) and Clorophyta (*U. lactuca*), with different levels of cellulose content in their tissues and a variety of phycocolloids (i.e., alginates (*F. Vesiculosus)*, carrageenan’s (*C. crispus)* and ulvan (*U. lactuca*). To remove most adsorbed MPs the samples were first washed with a surfactant (Triton X-100 (1 %, v/v)). Then two sequential steps were carried out for seaweed digestion. The first step was an incubation at 50 °C with a cellulase solution for 24 h, to degrade seaweeds cellulose matter. The second step involved an incubation period of 24 or 48 or 72 h at 65 °C with Hydrogen Peroxide solution (30 % v/v) (Fisher Chemical) (H_2_O_2_) to complete organic matter digestion [26,27] (Fig. 2).

**Fig. 3:**
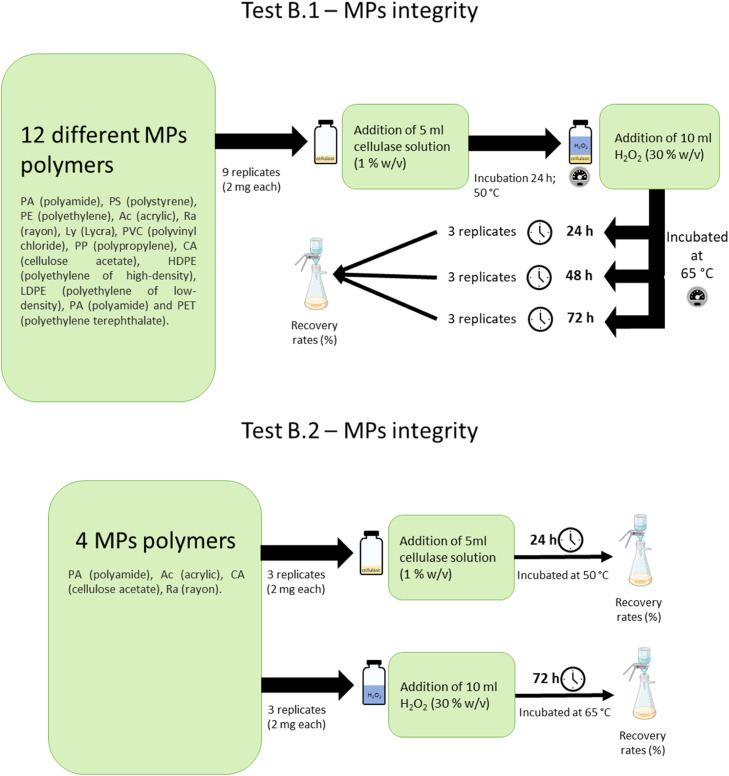
Representation of the tests performed, test B.1 on 12 different MPs polymers and tests B.2 on the 4 polymers that presented recovery rates lower than 90 %.
iii) Tests B.2 were performed with all MPs polymers that showed recovery rates lower than 90 %, to assess which reagent was responsible for polymer degradation, or if a combined effect of both reagents was responsible for the degradation observed. These consisted of 2 separate tests: one with an incubation at 50 °C with a cellulase solution for 24 h, and another with an incubation at 65 °C with H_2_O_2_ for 72 h (Fig. 3);

For every condition and MPs and seaweed species, 3 replicates were prepared (Figs. 2, 3).

The following methodology was used:

- Tests A: 10–15 g (fresh weight) of each seaweed species was used per sample. Before the digestion methodology, seaweed samples were washed in a Triton X-100 solution (1 % v/v) at constant mixing for 30 min to remove possible adsorbed MPs. The washing water was then filtered in a glass filtration system through a 0.45 μm pore size nitrate cellulose membrane filter which was transferred to a clean glass petri and dry at room temperature. The washed seaweeds were then dried at 60 °C for 24 h (temperature commonly used to dry seaweeds and a temperature that does not comprise MPs integrity [24]). After dried, the washed seaweeds were broken manually to homogenize the sample, and 3 sub-replicates of 0.5 g each were taken from the original sample and placed in 250 ml glass bottles as described in Fig. 2;
- Tests B.1 and B.2: 2 mg of each MPs type was used per replicate. MPs were weighed directly into previously cleaned and dried 26 ml scintillation glass vials.

After this, the methodology used was similar for both A and B.1 tests. Since the mass of sample (MP or seaweed) used varied between Tests A and B.1, the volumes of solution used were adapted (ensuring samples were completely submerged), keeping the same proportions and concentrations. For **Tests A**, 50 ml of 1 % (w/v) cellulase solution (0.5 g cellulase from *Aspergillus niger* (TCI Europe) diluted in 50 ml Acetate Buffer (0.1 M, pH 5.0) (ideal buffer for enzymatic activity) was added to each glass bottle and left to incubate at 50 °C (ideal temperature for enzymatic activity) for 24 h. Then, 100 ml H_2_O_2_ were added to each sample. Samples were then visually checked for complete digestion of seaweed tissue after 24, 48 or 72 h (Fig. 2) at 65 °C. This temperature was chosen as it has shown to be suitable for organisms tissues digestion [5,27]. Following each incubation period, samples were filtered in a glass filtration system, through a 0.45 um pore size nitrate cellulose membrane filter which was transferred to a clean glass petri and dry at room temperature. For **Test B.1**, 5 ml of 1 % (w/v) cellulase solution (0.05 g cellulase from *A. niger* (TCI Europe) diluted in 5 ml Acetate Buffer (0.1 M, pH 5.0) was added to each vial, ensuring the MPs were completely submerged. The vials were then left to incubate at 50 °C for 24 h. After this, 10 ml of H_2_O_2_ were added to the vial and different incubation periods were tested (i.e., 24, 48 or 72 h) at 65 °C (Fig. 3). After each incubation period, samples were filtered in a glass filtration system, through a 0.45 μm pore size nitrate cellulose membrane filter which was transferred to a clean glass petri and dry at room temperature. For **Test B.2**: cellulase effect: 5 ml of 1 % (w/v) cellulase solution (0.05 g cellulase from *A. niger* (TCI Europe) diluted in 5 ml Acetate Buffer (0.1 M, pH 5.0)) was added to each vial, ensuring the MPs were completely submerged. The vials were then left to incubate at 50 °C for 24 h; H_2_O_2_ effect: 10 ml of H_2_O_2_ were added to the vial during an incubation period of 72 h at 65 °C After each incubation period, the samples were filtered in a glass filtration system, through a 0.45 μm pore size nitrate cellulose membrane filter which was transferred to a clean glass petri and dry at room temperature (Fig. 3).

Filters obtained from the tests were then analyzed under a stereomicroscope: for test A to assess digestion efficiency of seaweed tissues; for test B.1 and B.2 to assess any change in color, size or general aspect of the MPs. MPs from test B.1 and B.2 were weighed before and after the digestion method to calculate recovery rates. Tested MPs were also analyzed by FTIR for polymer characterization before and after being subjected to the optimized methodology to ensure reliable identification of these polymers after exposure to the optimized methodology.

### Statistical analysis

A nested two-way ANOVA was used to assess how both incubation periods and MPs types affected the recovery rates of MPs after the digestion methodology with factor “Duration” nested in “MPs type“. Nested ANOVA and post-hoc analysis were performed using the aov and TukeyHSD functions in R from the native stats library.

## Method validation

(Results and Discussion are not part of a methods article. However, if possible, please provide data that validate the method.)

### Seaweed samples

**Fig. 4:**
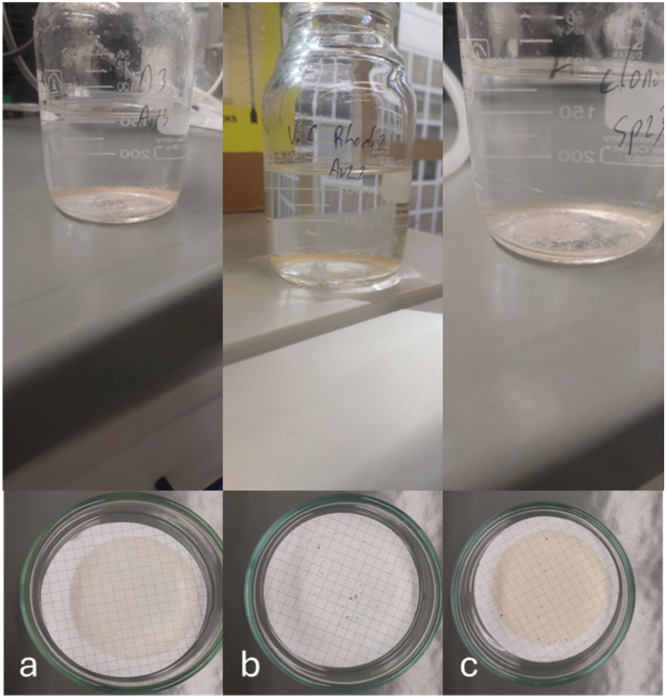
Digested samples resulting from the methodology described above, for 0.5 g of dry seaweed samples of (a) Phaeophyceae – F. vesiculosus, (b) Rhodophyta – C. crispus, (c) Clorophyta – U. lactuca. In all samples, only 1 filter was necessary to filter the digested samples.

Test A examined the digestion efficiency on 3 different seaweed species (*F. vesiculosus, C. crispus* and *U. lactuca*) with different levels of cellulose content and a variety of phycocolloids (i.e., alginates (*F. vesiculosus)*, carrageenan’s (*C. crispus)* and ulvan (*U. lactuca*)). The samples were successfully digested, the filters were clean with low levels of organic matter remaining, and only one 0.45 μm pore size cellulose nitrate membrane filter was necessary for filtration (Fig. 4). *F. vesiculosus* and *C. crispus* required 72 h of exposure to H_2_O_2_ for complete digestion, while *U. Lactuca* was completely digested after only 48 h of exposure.

For each seaweed tested, 3 replicates of 0.5 g dry weight were digested, totaling 9 samples. Afterwards, the optimized methodology was applied and, a total of 30 MPs were found across the 3 seaweed species (approx. 6.67 MPs per gram of dry weight). Of the MPs found, 29 were fibers and 1 was a particle, including blue, black, colorless, red and green colors, and sizes ranging from 0.4 mm and 1.0 mm. Regarding the sub-replicates performed for the seaweed digestion step, no significant differences in MPs concentration were observed between the different seaweed groups ((Kruskal-Wallis, *H* = 3.5963 *p* = 0.1656)). These results indicate that the proposed methodology efficiently digested seaweed tissues from different taxonomic groups using a single filtration step and worked consistently in different seaweeds with contrasting cellulose content and phycocolloid composition.

### Microplastics tests

**Fig. 5:**
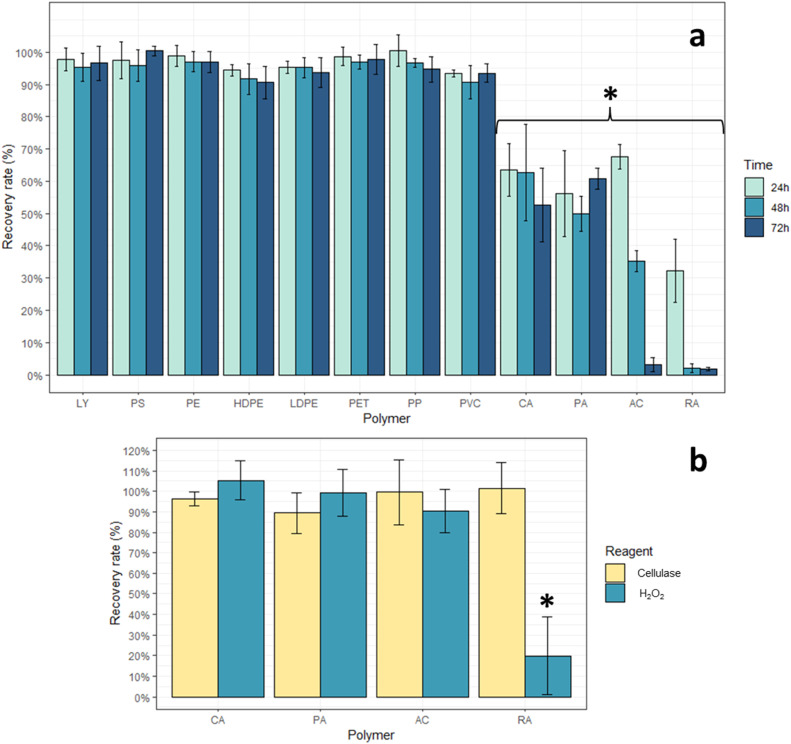
(a) Recovery rates ( %) (mean ± SD, *n* = 3) for all MPs polymers subjected to the digestion methodology for a period of incubation of 24 h at 50 °C in cellulase solution followed by 24, 48 or 72 h of exposure to H_2_O_2_ at 65 °C: MPs: lycra (Ly), polystyrene (PS), polyethylene (PE), polyethylene of high-density (HDPE), polyethylene of low-density (LDPE), polyethylene terephthalate (PET), polypropylene (PP), polyvinyl chloride (PVC), cellulose acetate (CA), polyamide (PA), acrylic (AC), rayon (RA); (b) Recovery rates ( %) (mean ± SD, *n* = 3) for the four polymers CA, PA, AC, RA when subjected to the methodology reagents separately: Cellulase solution (24 h incubation at 50 °C) and H_2_O_2_ (72 h incubation at 65 °C). (*) significant differences.

Most MPs types exposed to the methodology had recovery rates > 90 % regardless of the incubation time (Fig. 5a). The exceptions were AC, RA, PA and CA which showed significantly lower recovery rates (*F* = 145.909, *p* < 0.001) (Fig. 5a, Table 1). After a 24 hour H₂O₂ incubation, the lowest recovery rate was 32 % for RA. After 48 and 72 h H₂O₂ incubation, this polymer was almost completely degraded. Tests B.2 with reagents used separately, showed recovery rates ≥ 100 % with cellulase solution incubation only, and significant lower recovery rates when using only H₂O₂ (*F* = 13.22, *p* < 0.001) (Fig. 5b). This differs from results observed in other methodologies that utilize H₂O₂, where no loss in recovery rate of RA was observed [5,27], and can be explained by the MPs used in this work not being brand new. In tests B.1, AC presented significant differences between incubation time for 24 and 48h/72 h (*F* = 2.984, *p* < 0.001), with recovery rates > 65 % at 24 h but almost 0 % recovery rate at 72 h (Fig. 5a, Table 1). Tests B.2 showed recovery rates ≥ 90 % when reagents were used separately (Fig. 5b), which might indicate that degradation occurs from a combined effect of both reagents. In tests B.1, PA and CA showed no significant differences across incubation times (*F* = 2.984, *p* > 0.05), maintaining recovery rates > 50 % (Fig. 5a, Table 1). The uniform recovery rates across varying recovery times imply that the lower recoveries observed may be attributable to the degradation of a plastic surface additive during the methodology, given the absence of FTIR spectral changes. In fact, FTIR analysis showed no changes in spectra obtained independently of recovery rates observed, and all polymers were still identifiable. For each studied polymer detailed recovery ( %) data, incubation time and reagent used (Tests B.1 and B.2; mean ± SD, *n* = 3) are presented in Fig. 5 and Table 1. Overall, the MPs tests confirm that the optimized digestion methodology preserves mass and FTIR-identifiable structure of most non-cellulose-based polymers (recovery > 90 %), while having some limitations in recovery for cellulose-based (RA, CA), AC and PA polymers. Results should be interpreted with caution when these late materials are expected in environmental samples. Other studies have shown degradation of PA when using H_2_O_2_ (30–35 % v/v) as our study [28], however, to the best of our knowledge, no other study with cellulase based enzymatic digestion evaluate the possible degradation of the polymers AC, RA and CA.

**Table 1:** Results obtained in the different tests performed with 12 MPs polymers, different seaweed groups at different periods of incubation with cellulase solution for 24 h and H_2_O_2_ 30 % solution for 24, 48 and 72 h, as well as tests using the methodology reagents separately, with the 4 polymers (AC, RA, PA, CA) showing significant lower recovery rates. MPs polymers: AC (acrylic), LY (Lycra), PE (polyethylene), PS (polystyrene), RA (rayon), PA (polyamide), CA (cellulose acetate), HD-PE (polyethylene of high-density), LD-PE (polyethylene of low-density) and PET (polyethylene terephthalate), PP (polypropylene), PVC (polyvinyl chloride). Red sign = major changes; yellow sign = minor changes; green sign = no changes.

**Fig. 6:**
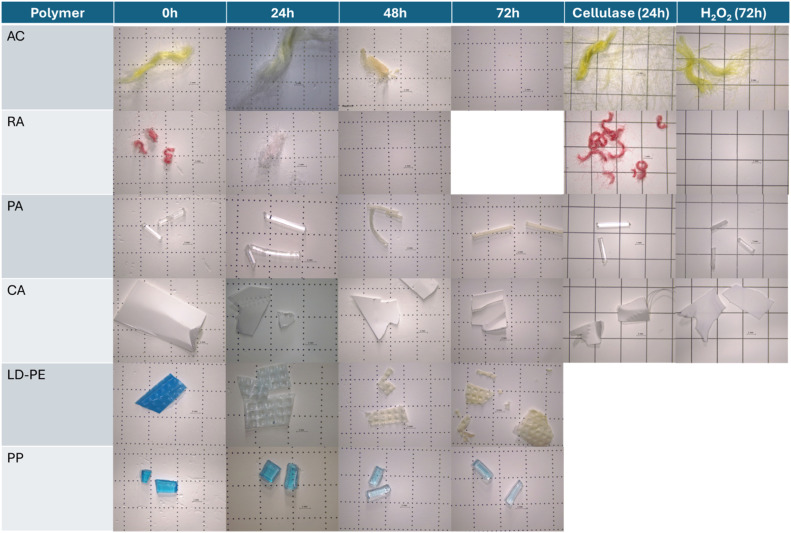
MPs polymers that presented loss of color/weight or change in aspect after the digestion methodology after 24, 48 or 72 h exposed to H_2_O_2_ at 65 °C, as well as exposure to the used reagents separately cellulase solution for 24 h at 50 °C as well as only H_2_O_2_ for 72 h at 65 °C.

Some MPs types such as PP showed some loss of color with the increase in incubation time, and LDPE completely lost color after 48 h of incubation time (Fig. 6). For each MPs type with recovery rates > 90 %, no significant differences in their recovery rates were observed between the three incubations times (*F* = 2.984, *p* > 0.05) (Fig. 5a). From these, most MPs types showed no changes in color, weight or visual aspect (Table 1). MPs with significant lower recovery rates (Fig. 5a), such as AC and RA showed some loss of color with the increase in incubation time, and PA became opaquer, while CA became more brittle (Fig. 6, Table 1).

## Final considerations

The methodology optimized in this study proved efficient in digesting seaweed with different cellulose composition and a variety of phycocolloids for MPs extraction. Although some methodologies perform an extra incubation step with the addition of protease [1,12] to degrade the resulting proteins of the enzymatic digestion with cellulase, our results showed that this was not necessary to get acceptable results, since filters were clean and suitable for further MPs analyze. Skipping this step considerably reduces the time period of the methodology, reduces hands-on labor enabling parallel processing of multiple samples, lowers costs of digestion per sample and might reduce MPs exposure to possible degradation and/or loss.

This methodology was also able to maintain all MPs characteristic of six of the 12 MPs tested, including the most commonly found in the environment such as PE, PP, PS (Table 1). However, cellulose-based polymers as RA, was almost completely degraded during the methodology in the 72 h incubation time. RA degradation was expected since the cellulase use in the early phase of the methodology directly attacks the β−1,4 glycosidic bonds in the cellulose structure of RA. The combination with the oxidative degradation caused by H_2_O_2_ lead to extensive polymer breakdown. For PA, while cellulase does not directly affect this polymer, H_2_O_2_ attacks the amide bonds (-CO—NH-) through oxidation, leading to the degradation of this polymer as observed in our study and others studies. However, this degradation is only observed through the combined effect of both reagents used in this methodology (cellulase solution and H_2_O_2_). As observed in our Tests B.2 results, no degradation was observed for this polymer when only one of the reagents is used. Other studies that employ a digestion methodology only based on H_2_O_2_ also did not observe any degradation [1,28]. All polymers were still identifiable through FTIR analysis, with identical spectra even in cases where recovery rates were lower than 90 %.

Final protocol is shown below:

1. All material should be cleaned using ethanol 96 % and filtered deionized water, all material should be covered with aluminum foil when not in use, a glass petri with filtered deionized water should be use to assess airborne contamination during the different steps of the procedure, a procedural blank with deionized water that it is treated as a sample should be included;
2. Seaweed should be placed in a beaker to be mixed and washed in a Triton X-100 solution (1 % v/v) for 30 min to remove adsorbed MPs with the help of a wash bottle filled with the same solution;
3. The resulting solution should then be filtered through a 0.45 μm pore size nitrate cellulose membrane filter to assess seaweed contamination through adsorption.
4. The washed seaweed should be left to dry at 60 °C until completely dried;
5. When dried, the seaweed should be manually grinded as fine as possible, homogenized, and 0.5 g should be transferred into a 250 ml glass bottle;
6. 50 ml of 1 % (w/v) cellulase solution (0.5 g cellulase from *a. niger* diluted in 50 ml acetate buffer (0.1 M, pH 5.0)) should be added to the glass bottle and covered, but not closed, to prevent any contamination (e.g., aluminum foil).
7. Samples are then placed in the oven at 50 °C for 24 h and manually agitated periodically;
8. After 24 h, add 100 ml 30 % (v/v) H_2_O_2_ solution and place samples in the oven at 65 °C for a maximum of 72 h, while manually agitated periodically; if the seaweed is satisfactorily digested before 72 h the samples should be removed;
9. After digestion, the sample can be filtered through a glass filtration system using a 0.45 μm pore size nitrate cellulose membrane filter, the filtration system as well as the glass bottle should be rinsed several times with filtered deionized water to ensure all material is clean;
10. Filters should be left to dry at room temperature, and visually inspected for the presence of MPs using a stereomicroscope;
11. MPs should be characterized by type of particle, color, size and their polymer whenever possible (e.g., analysis by FTIR, Raman spectroscopy…).

## Limitations

Despite being the only way to successfully digest seaweed organic matter, enzymatic-oxidative methodology using cellulase and H₂O₂ might not be suitable for the quantitative monitoring of rayon (RA) and acrylic (AC) particles, as these materials undergo significant mass loss during long incubation periods Therefore, results for these polymers derived from this protocol should be interpreted as underestimates only. For cellulose acetate (CA) and polyamide (PA), the protocol produced partial losses (53 % and 61 % recovery respectively), which may reflect the degradation of surface layers/additives, as FTIR identifiability remains. These polymers should therefore be reported with explicit caution statements. A key limitation is synergistic degradation from the enzymatic and oxidative steps (mandatory for the successful digestion of seaweed organic matter). MPs recoveries were substantially higher when cellulase or H₂O₂ were applied separately than when applied sequentially, indicating that the two-step design increased polymer susceptibility. To the best of our knowledge, no other study using cellulase as part of the digestion methodology tested these polymers (RA, AC, CA, PA) for degradation and/or loss of weight.

Visible alterations (e.g. color loss in PP/LDPE; opacity in PA; and brittleness in CA) may lead to misidentification and/or fragmentation during visual counting. Therefore, minimizing incubation to the shortest digestion time necessary and confirming polymers composition is recommended.

## Ethics statements

Not applicable.

## CRediT author statement

R. Pereira: Conceptualization, Methodology, Validation, Formal analysis, Investigation, Writing – original draft, Writing – review & editing. C. Marisa R. Almeida: Validation, Writing – review & editing Supervision, Project administration, Funding acquisition. S. Ramos: Validation, Writing – review & editing Supervision, Project administration, Funding acquisition.

## Declaration of competing interest

The authors declare that they have no known competing financial interests or personal relationships that could have appeared to influence the work reported in this paper.

## Data Availability

Data will be made available on request.
